# Epigenetic Regulation and Therapeutic Targeting of Alternative Splicing Dysregulation in Cancer

**DOI:** 10.3390/ph18050713

**Published:** 2025-05-12

**Authors:** Yan Lei, Maode Lai

**Affiliations:** 1 Department of Pharmacology, China Pharmaceutical University, Nanjing 210009, China; ly858564859@163.com; 2 Research Unit of Intelligence Classification of Tumor Pathology and Precision Therapy, Chinese Academy of Medical Science (2019RU042), Key Laboratory of Disease Proteomics of Zhejiang Province, Department of Pathology, Zhejiang University School of Medicine, Hangzhou 310058, China; 3 Department of Pathology, Sir Run Run Shaw Hospital, Zhejiang University School of Medicine, Hangzhou 310016, China

**Keywords:** alternative splicing, epigenetic modifications, cancer, therapeutic strategies

## Abstract

Alternative splicing enables a single precursor mRNA to generate multiple mRNA isoforms, leading to protein variants with different structures and functions. Abnormal alternative splicing is frequently associated with cancer development and progression. Recent studies have revealed a complex and dynamic interplay between epigenetic modifications and alternative splicing. On the one hand, dysregulated epigenetic changes can alter splicing patterns; on the other hand, splicing events can influence epigenetic landscapes. The reversibility of epigenetic modifications makes epigenetic drugs, both approved and investigational, attractive therapeutic options. This review provides a comprehensive overview of the bidirectional relationship between epigenetic regulation and alternative splicing in cancer. It also highlights emerging therapeutic approaches aimed at correcting splicing abnormalities, with a special focus on drug-based strategies. These include epigenetic inhibitors, antisense oligonucleotides (ASOs), small-molecule compounds, CRISPR–Cas9 genome editing, and the SMaRT (splice-switching molecule) technology. By integrating recent advances in research and therapeutic strategies, this review provides novel insights into the molecular mechanisms of cancer and supports the development of more precise and effective therapies targeting aberrant splicing.

## 1. Introduction

A new splicing model, alternative splicing, was introduced by Gilbert in 1978, explaining how it is possible for one gene to produce multiple mRNAs [1], creating multiple distinct proteins and increasing human transcriptome complexity. This theory significantly expanded our understanding of gene expression regulation. It has been demonstrated that alternative splicing occurs in about 95% of eukaryotic genes with multiple exons, as shown by mRNA-Seq and EST-cDNA data [2]. This finding underscores the ubiquity and functional importance of alternative splicing as a critical regulatory mechanism.

**Figure 1:**
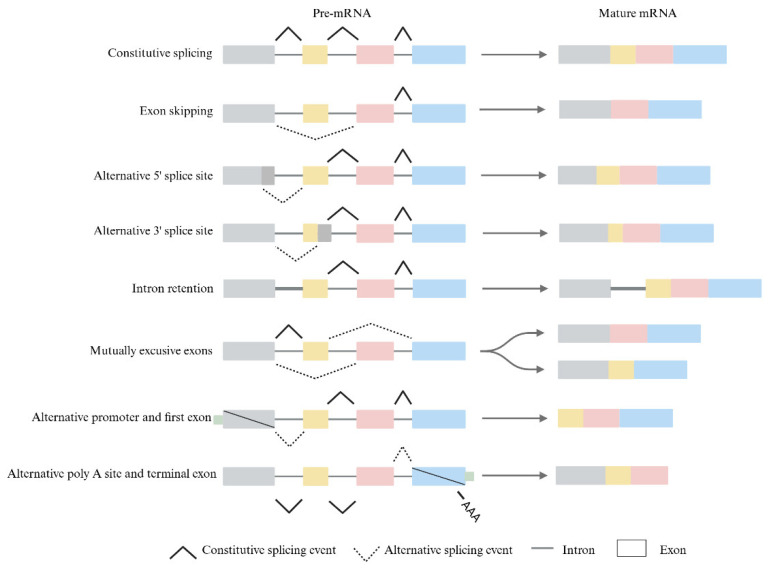
Diagram of alternative splicing model classifications: Alternate splicing consists of exon skipping, alternative splice sites, intron retention, mutually exclusive exons, alternative promoters, alternative Poly A sites, and alternative terminal exons.

As a normal splicing process, constitutive splicing removes introns from pre-mRNA [3]. On the other hand, alternative splicing generates different functional transcripts from the pre-mRNA, which can be categorized by their splicing patterns into seven different types [4,5]: (1) Exon Skipping: As a result of cis-regulatory elements and trans-acting factors, exons in the middle of the transcript are removed from the final transcript. (2) Alternative 5′ Splice Site: A different 5′ splice site changes the upstream boundary of an exon in the final transcript. (3) Alternative 3′ Splice Site: A different 3′ splice site changes the downstream boundary of an exon in the final transcript. (4) Intron Retention: Introns are retained along with adjacent exons in the final transcript. (5) Exon Exclusion: Two exons compete for inclusion in the final transcript and only one will be retained. (6) Alternative Promoters: A different initiating exon of the transcript is selected. (7) Variable Terminator: A different terminating exon of the transcript is selected (Figure 1). Different patterns of alternative splicing produce distinct transcripts, which can affect gene expression and mRNA translation into proteins, resulting in various functional properties and a greater diversity of the human transcriptome [6,7]. The regulatory mechanisms of alternative splicing are complex and influenced by various factors. As research progresses, it is likely that new splicing types will be discovered.

Alternative splicing is highly complex in terms of its mechanisms and regulation. Earlier studies based on electron microscopy images revealed that co-transcriptional splicing occurs in Drosophila embryos [8]. Subsequent research demonstrated that splicing and transcription occur simultaneously and are an inseparable dynamic process. In eukaryotes, alternative splicing facilitates genetic information transfer and is a signaling pathway target [9]. Chromatin structure and transcription factors are involved as well as the splicing machinery. These studies provide us with a theoretical framework for understanding the coupling of transcription and splicing, emphasizing the dynamic nature of this process and its multi-layered regulatory mechanisms. In *Saccharomyces cerevisiae*, co-transcriptional recruitment of U1 snRNP occurs in genes with short terminal exons, and post-transcriptional recruitment of U2 snRNP occurs in genes with long terminal exons [10,11]. Their research further deepens our understanding of the mechanisms underlying the interaction between splicing factors and transcription factors. A variety of factors influence pre-mRNA splicing, including histone modification within chromatin [9,12]. These studies provide important insights into the complex mechanisms of alternative splicing, particularly regarding the coupling of transcription and splicing, the dynamic process of splicing factor recruitment, and the role of chromatin structure in splicing.

The regulation of alternative splicing is intricate and can be divided into three primary components: cis-acting elements, trans-acting factors, and the structure of chromatin, along with various epigenetic modifications [13]. This process is influenced not only by the RNA sequence itself but also by the multiple regulatory interactions of various molecular factors within the cell. Epigenetics is essential for alternative splicing, as it modulates the chromatin structure to produce diverse phenotypes without affecting the genetic sequence. Epigenetic factors encompass DNA methylation, histone modifications, and nucleosome positioning [14]. Chromatin remodeling, non-coding RNAs (ncRNAs), and m6A modifications are also considered epigenetic factors [9,15,16]. Disruption of epigenetics and alternative splicing is highly associated with cancer. Dysregulation of alternative splicing has emerged as a key factor in cancer development, becoming an increasingly important focus in cancer research. Epigenetic modifications not only play a role at the transcriptional level but also further regulate gene expression post-transcriptionally by influencing the splicing process. The interplay between these mechanisms not only deepens our understanding of the molecular basis of cancer but also holds potential for identifying new targets for early diagnosis and therapeutic intervention. Although numerous studies have supported the existence of links among epigenetic modifications, dysregulation of alternative splicing, and cancer development, the precise mechanisms underlying this relationship remain incompletely understood. For example, different types of epigenetic modifications and splicing dysregulation in cancer may play distinct roles, and the patterns of splicing alterations can vary across different tumor types. Accurately distinguishing and interpreting these complex interactions requires further investigation through cellular biology and clinical research. Moreover, since epigenetic modifications and splicing dysregulation are likely interwoven, intervening in this intricate network for therapeutic purposes remains a significant challenge.

**Figure 2:**
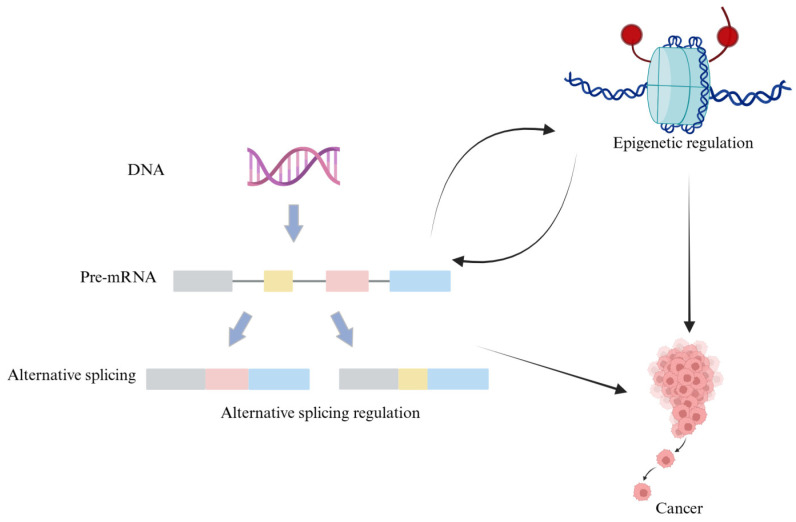
The relationships among cancer, epigenetics, and alternative splicing.

In this review, we focus on the therapeutic potential of targeting epigenetics and alternative splicing in cancer treatment (Figure 2). We discuss how epigenetic modifications regulate RNA splicing patterns and gene expression, highlighting their implications for cancer progression. Given that splicing abnormalities in cancer cells are closely linked to epigenetic dysregulation, targeting these pathways presents a promising therapeutic strategy. We emphasize the roles of RNA-binding proteins, non-coding RNAs, and other molecular regulators as druggable targets. Furthermore, we provide an in-depth review of emerging pharmacological interventions, including small molecule inhibitors, antisense oligonucleotides (ASOs), and CRISPR–Cas9 based approaches, which are currently in clinical trials and show potential for correcting aberrant splicing events in cancer cells. By integrating recent advancements in drug development, this review underscores the potential of epigenetic and splicing-targeted therapies in advancing precision oncology. Ultimately, the development of effective therapeutic strategies aimed at modulating these molecular mechanisms may lead to more precise and improved cancer treatment outcomes.

## 2. How Epigenetics Affects Alternative Splicing

### 2.1. Dive into DNA Methylation’s Role in Alternative Splicing

**Figure 3:**
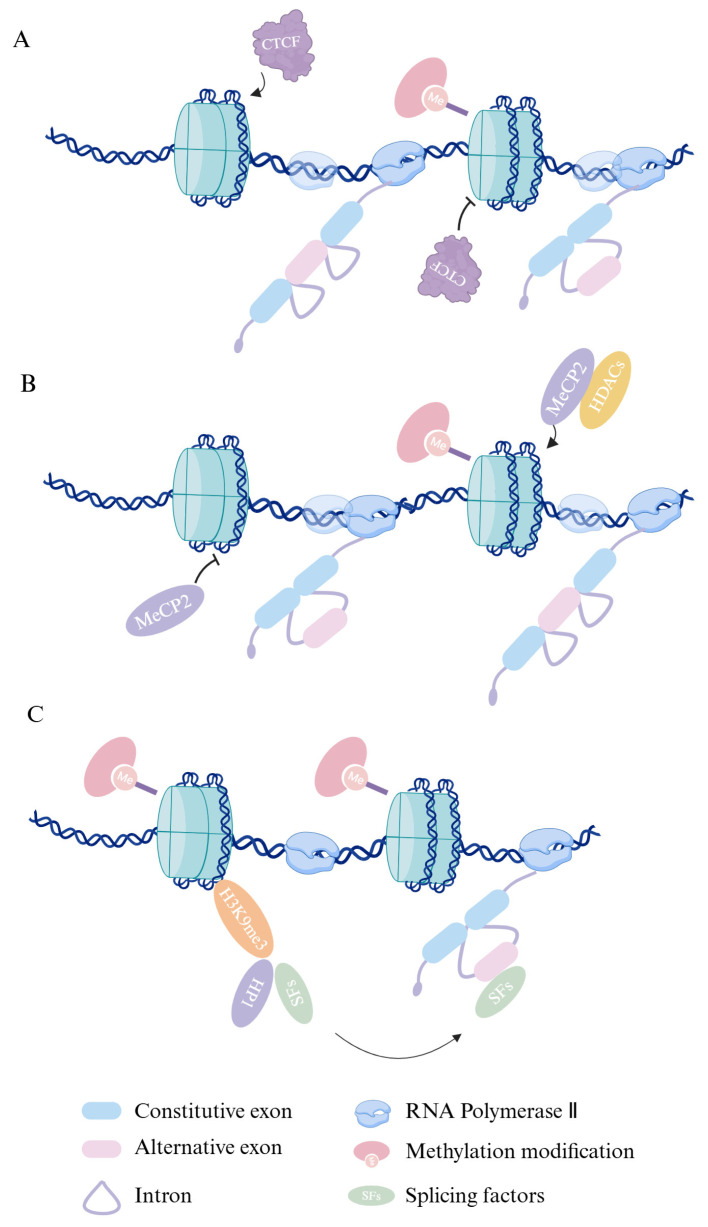
The mechanisms by which DNA methylation influences alternative splicing. (**A**) When DNA is methylated, CTCF binding is inhibited, leading to reduced Pol II elongation rates and increased exon skipping. (**B**) When DNA is methylated, the methylation-binding protein MeCP2 binds to the methylated sequences and recruits HDAC enzymes, thereby slowing Pol II elongation rates and promoting exon inclusion. (**C**) When DNA is methylated, the H3K9me3-recognizing protein HP1 recruits splicing factors (SFs), causing them to transfer to pre-mRNA during transcription, leading to exon skipping.

DNA methylation exerts a pivotal influence in the field of epigenetics by altering gene expression profiles through its modulation of the binding affinity of transcription factors and other DNA-binding proteins. Research has shown that DNA methylation might play a role in modulating the regulation of alternative splicing. Exon splice sites exhibit higher DNA methylation levels than their adjacent introns, with DNA methylation regulating 22% of alternative exon splicing [17]. This underscores the critical role of DNA methylation in the regulation of splice site selection. For instance, DNA methylation may impact the rate of Pol II elongation, influencing alternative splicing. The DNA-binding protein CTCF (CCCTC-binding factor) can enhance the inclusion of weak upstream exons by facilitating Pol II-mediated alternative splicing in mammals by binding to specific DNA sequences [18] (Figure 3A). Additionally, the multifunctional protein methyl-CpG-binding protein 2 (MeCP2) regulates alternative splicing by modifying Pol II elongation kinetics, a process facilitated by its recruitment of various histone deacetylases [19,20] (Figure 3B). Interaction with heterochromatin protein 1 (HP1) accounts for 20% of the overall impact of DNA methylation on splicing. Genome-wide analyses revealed that the three HP1 isoforms, when individually or collectively inhibited, are capable of regulating alternative splicing [17,21,22] (Figure 3C). Taken together, DNA methylation not only influences gene expression by modulating the binding affinity of transcription factors to DNA, but also plays a crucial role in regulating alternative splicing. The growing understanding of DNA methylation’s involvement in splicing regulation offers promising avenues for the development of epigenetic therapies, particularly in the context of diseases where splicing dysregulation contributes to pathology such as cancer. As epigenetic drugs targeting DNA methylation advance, their potential for correcting splicing defects presents an exciting opportunity for therapeutic intervention.

### 2.2. Histone Modifications and Their Impact on Alternative Splicing

**Figure 4:**
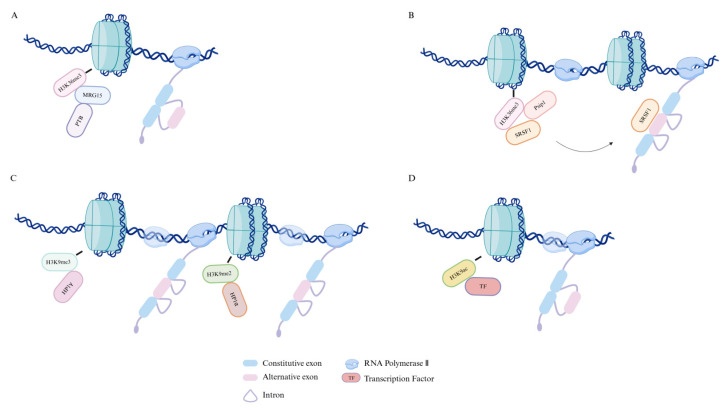
How histones influence alternative splicing. (**A**) MRG15 interacts with H3K36me3 and brings the splicing silencer protein PTB to specific RNAs, thereby inhibiting exon inclusion. (**B**) Psip1, by binding to H3K36me3, can localize to the nascent RNA and recruit the splicing regulator SRSF1 to affect exon inclusion. (**C**) H3K9me2 and H3K9me3, by binding to HP1α and HP1γ, respectively, inhibit Pol II transcriptional elongation, thereby increasing alternative exon inclusion. (**D**) Hyperacetylation of H3 histones leads to a less condensed chromatin structure, increasing Pol II elongation rates and facilitating exon skipping.

Histone modifications also exert a significant impact on alternative splicing. Genome-wide analyses revealed that nucleosomes, marked by several histone modifications, are non-randomly enriched in exons [23]. Notably, H3K36me3 is particularly enriched in constitutive exons [24,25]. Recent research indicates that in *Schizosaccharomyces pombe*, H3K36me3 marks are enriched in exons [26], which may further suggest that the transition between regions of H3K36me3 enrichment and depletion could serve as a marker for exon/intron boundaries. This theory provides a compelling model for the relationship between histone modifications and alternative splicing. H3K36me3 is also detected by the MORF-related gene 15 (MRG15) protein in mesenchymal cells, which recruits splicing silencer protein polypyrimidine tract-binding (PTB) to its target RNA, promoting the skipping of alternative exons [23] (Figure 4A). Psip1/p52, through its bromodomain, binds the H3K36me3 mark and modulates alternative exon inclusion by recruiting the splicing regulatory factor SRSF1 [27] (Figure 4B). These studies further elucidate the critical role of histone modifications in alternative splicing, specifically through their interactions with distinct splicing factors to control exon inclusion or skipping.

Additional research has demonstrated that the regions of chromosome containing variable exons of CD44 are highly enriched with the histone modification H3K9me3. This mark is recognized by HP1γ, which promotes alternative exon inclusion by reducing local transcription elongation rates [22]. Further studies indicated that siRNA-mediated targeting of sequences near the exons, which affects local H3K9me2 marking, can regulate exon splicing. The heterochromatin mark H3K9me2 is recognized by the heterochromatin protein HP1α, which, in turn, influences transcription elongation and facilitates the inclusion of exon EDI [28] (Figure 4C).

Additionally, neuronal depolarization triggers the skipping of exon 18 in neural cell adhesion molecule (NCAM) mRNA and results in the restriction of H3K9 hyperacetylation to the internal region of another exon within the NCAM gene. H3K9ac has also been demonstrated to increase Pol II elongation rates (Figure 4D). Upon removal of depolarization, this effect can be replicated and further potentiated by the histone deacetylase inhibitor trichostatin A (TSA) [29]. These findings provide insight into how alternative splicing can be regulated by histone modifications, influencing Pol II elongation rates. Taken together, these insights affirm the therapeutic promise of targeting histone modification pathways to modulate alternative splicing. With several histone-modifying enzymes already serving as drug targets in cancer and other diseases, understanding their role in RNA processing may guide the development of more refined epigenetic therapies with splicing-specific effects.

## 3. Interplay Between Alternative Splicing and Epigenetics

There is an interactive interplay between epigenetics and splicing regulation. Research has shown that in primary neurons and mouse embryonic stem cell-derived neurons, the RNA-binding protein Hu interacts with histone deacetylase 2 in an RNA-dependent manner, inhibiting its deacetylase activity to regulate alternative splicing [30]. The spliceosome inhibitor spliceostatin A, which mimics mutations at splice sites, suppresses the efficiency of alternative splicing and leads to a rapid repositioning of H3K36me3 from the 5′ end to the 3′ end [31]. This suggests that splice regulation is not only directly controlled by RNA but is also profoundly influenced by epigenetic modifications, highlighting a potential crosstalk between alternative splicing and histone modifications.

In addition to its impact on histone modifications, alternative splicing can also influence DNA methylation by regulating the splicing of epigenetic factors. In embryonic stem cells, downregulation of PTBP1 promotes the retention of a co-transcriptionally activated intron in the mRNA encoding the DNA methyltransferase DNMT3B. The retention of this intron, driven by PTBP1, suppresses DNMT3B production, thereby ensuring proper DNA methylation of neuronal genes [32]. This mechanism provides a rationale for considering splicing factors as upstream regulators of epigenetic enzymes, expanding the spectrum of druggable targets for diseases such as cancer and neurodevelopmental disorders.

Furthermore, histone modifications and DNA methylation interact in a complex manner. Studies have shown that in AML patient samples and cell lines, hypermethylation of DNA is associated with the loss of H3K4me3 and the acquisition of unmethylated H3K4 [33]. Methylated DNA, when incorporated into chromatin, binds to the transcriptional repressor MeCP2, which in turn recruits histone deacetylases to suppress transcription [34]. Conversely, histone modifications also exert a significant influence on DNA methylation; for instance, the binding activity of H3K9me2/3 is essential for UHRF1-mediated DNMT1 to facilitate DNA methylation [35]. HP1subtypes HP1α and HP1β have been shown to bind to methylated H3K9 residues [36,37,38]. Additionally, the DNA methylation of pericentric satellite repeats by DNMT3A and DNMT3B is dependent on a functional Suv39h–HP1 histone methylation system [39].

Together, these findings highlight a tightly regulated and reciprocal network between splicing, histone modifications, and DNA methylation, all of which contribute to the fine-tuning of gene expression. From a pharmaceutical standpoint, targeting these epigenetic–splicing interactions offers promising strategies for therapeutic development, particularly in malignancies where these regulatory axes are frequently dysregulated. Future drug design efforts may benefit from incorporating dual-acting agents that can simultaneously modulate the chromatin state and RNA processing dynamics to enhance clinical efficacy.

## 4. Epigenetic Regulation’s Influence on Alternative Splicing in Cancer

Increasing evidence underscores the critical role of alternative splicing in cancer pathogenesis, with potential mechanisms involving dysregulated proliferation and epigenetic alterations that drive disease initiation and progression [40]. Although epigenetic alterations do not directly change the DNA sequence, they can promote genomic instability and disrupt DNA repair mechanisms, thereby inducing gene mutations and influencing various cancer-related cellular processes, such as apoptosis, angiogenesis, tumor invasion, and metastasis [41]. For example, hypermethylation of mismatch repair genes like MLH1 leads to microsatellite instability and the accumulation of mutations—a known pathway in colorectal and other cancers [42]. Moreover, loss of Polycomb Repressive Complex 1/2 (PRC1/2) components has been shown to induce a persistent oncogenic state through chromatin remodeling and JAK–STAT pathway activation, even in the absence of permanent genetic mutations, as demonstrated in Drosophila models [43]. Comprehensive analysis of RNA-seq data across various cancer types [44] has uncovered widespread aberrant splicing events, many of which correlate significantly with patient survival [45]. For example, TP53, BCL-X, and CD44 undergo epigenetically regulated alternative splicing that alters protein function and contributes to tumor growth, immune evasion, and metastasis. In TP53, abnormal splicing can result in dominant-negative isoforms that interfere with wild-type tumor suppressor function. In BCL-X, histone modifications influence the balance between pro-apoptotic (BCL-XS) and anti-apoptotic (BCL-XL) isoforms, directly impacting cell survival [46,47,48]. Increasingly, studies have highlighted the potential of targeting alternative splicing as a promising therapeutic approach [49]. In preclinical models, mutations in the splice acceptor and donor sites of MET exon 14 lead to exon skipping, offering a new therapeutic strategy for 4% of patients with lung adenocarcinoma [50,51]. Therefore, alternative splicing is not only a molecular event but is closely linked to the biological advantages of tumor cells, including enhanced proliferation, metastasis, and resistance to treatment. The functional consequences of splicing alterations provide tumor cells with a selective advantage and may represent viable therapeutic targets.

A notable clinically validated example is the androgen receptor splice variant 7 (AR-V7), frequently detected in circulating tumor cells and peripheral whole blood of patients with metastatic castration-resistant prostate cancer (mCRPC). AR-V7 lacks the ligand-binding domain and remains constitutively active even in the absence of androgens. Multiple studies have demonstrated that AR-V7 expression is associated with poor clinical outcomes and resistance to androgen receptor signaling inhibitors (ARSIs) such as abiraterone and enzalutamide. As a result, AR-V7 is now considered both a prognostic and predictive biomarker in mCRPC, illustrating how specific splice variants can directly impact therapeutic efficacy and reflect cancer hallmarks like hormonal independence and treatment resistance [52,53]. The clinical relevance of AR-V7 exemplifies how splice variant profiling can inform precision medicine strategies in oncology.

Research has identified 260 exons in lung cancer that exhibit both differential methylation and varying expression levels, with the genes harboring these differentially regulated exons being significantly enriched in biological processes associated with cancer biomarkers, particularly in the regulation of angiogenesis [54]. This finding suggests that DNA methylation-dependent alternative splicing contributes to cancer progression. Additionally, epigenetic modifications, particularly those associated with chromatin regions enriched in H3K27ac, regulate the expression of alternative splicing variants in HPV-associated oropharyngeal squamous cell carcinoma, thereby promoting tumorigenesis [55]. These results underscore the role of epigenetically driven alternative splicing in cancer progression, presenting a novel therapeutic paradigm.

From a therapeutic standpoint, targeting epigenetic regulators that influence splicing decisions offers a novel and potentially synergistic strategy in oncology. Unlike traditional approaches focused on genetic mutations or bulk gene expression, epigenetic–splicing axis interventions allow for precise modulation of transcript variants that are functionally relevant to tumor biology. For example, small molecule inhibitors of histone deacetylases (HDACs), DNA methyltransferases (DNMTs), and splicing modulators could be employed alone or in combination to reprogram aberrant splicing profiles in cancer. This integrated therapeutic paradigm—rooted in epigenetic and post-transcriptional regulation—may unlock new avenues for the development of personalized and isoform-specific cancer treatments.

## 5. Therapeutic Approaches Targeting Epigenetics and Alternative Splicing

Epigenetic dysregulation and aberrant alternative splicing are intimately associated with the onset and progression of numerous human diseases, particularly cancer. Unlike permanent genetic mutations, epigenetic changes are reversible, making them attractive therapeutic targets for small molecule interventions. A growing class of epigenetic drugs, including histone deacetylase inhibitors (HDACis), DNA methyltransferase inhibitors (DNMTis), and bromodomain and extraterminal domain (BET) inhibitors, has been developed to modulate the chromatin architecture and influence splicing outcomes, offering promising avenues for restoring gene expression homeostasis and correcting oncogenic splicing programs.

### 5.1. Histone Deacetylase Inhibitors (HDACis)

Several drugs targeting epigenetic modifications have been developed, including five histone deacetylase (HDAC) inhibitors that are currently approved for clinical use. These include vorinostat (SAHA), approved in 2006 for treating cutaneous T-cell lymphoma; romidepsin (FK-228), approved in 2009 for the same indication; belinostat (PXD-101), approved in 2014 for the treatment of relapsed or refractory peripheral T-cell lymphoma; panobinostat (LBH589), approved in 2015 for the treatment of multiple myeloma; and chidamide, developed in China and approved in 2015 for relapsed or resistant peripheral T-cell lymphoma (Table 1). Additionally, valproic acid (VPA) acts similarly to HDAC inhibitors such as trichostatin A by enhancing the acetylation of histones H3 and H4, which activates various exogenous and endogenous promoters [56]. Abexinostat (CRA-024781) is a novel, broad-spectrum HDAC inhibitor based on hydroxamic acid that has demonstrated antitumor activity in preclinical studies and is currently in phase I clinical trials for cancer [57]. The novel small molecule CUDC-101 simultaneously inhibits EGFR, HER2, and HDAC, showing preclinical activity in head and neck squamous cell carcinoma [58]. Pracinostat is a potent oral pan-HDAC inhibitor with activity in acute myeloid leukemia (AML) and demonstrates synergistic antitumor effects when combined with azacitidine [59]. Givinostat (ITF2357), a novel hydroxamic acid-derived HDAC inhibitor, demonstrates significant targeted anticancer activity while maintaining low toxicity [60,61,62]. Mocetinostat, a selective HDAC inhibitor targeting specific isotypes, promotes histone acetylation accumulation, cell cycle arrest, and apoptosis across multiple cancers [63,64].

Recent studies have shown that HDAC inhibitors, such as vorinostat (SAHA), can regulate the alternative splicing of key apoptotic genes like Bcl-X. Specifically, SAHA shifts the splicing of Bcl-X toward the pro-apoptotic Bcl-XS isoform, thereby promoting apoptosis in cancer cells [78]. This modulation of splicing may play an important role in addressing aberrant cancer splicing, which contributes to tumor cell survival and resistance to treatment. Aberrant splicing of genes like Bcl-X results in isoforms that favor tumor cell survival, and the ability of HDAC inhibitors to regulate these splicing events offers new opportunities to correct or exploit these splicing alterations. Therefore, combining splicing regulation with HDAC inhibition may represent a promising strategy to enhance therapeutic efficacy and overcome treatment resistance.

### 5.2. DNA Methyltransferase Inhibitors (DNMTis)

DNA methyltransferases (DNMTs), a conserved group of enzymes involved in cytosine methylation, play a crucial role in epigenetic regulation. DNMT inhibitors (DNMTis) can reversibly modulate abnormal DNA methylation. The FDA has authorized two nucleoside analogs, 5-azacytidine (azacitidine) and 5-aza-2′-deoxycytidine (decitabine), for treating myelodysplastic syndromes. Guadecitabine sodium (SGI-110), a dinucleotide incorporating 5-aza-2′-deoxycytidine, acts as a potent DNA methylation inhibitor that inhibits tumor growth [79]. The nucleoside analog NTX-301 (5-aza-4′-thio-2′-deoxycytidine or Aza-TdC) integrates into DNA and interacts with the active site of DNMT1, promoting demethylation and restoring the expression of tumor suppressor genes [80]. RX-3117, an additional nucleoside analog, reduces DNMT1 levels in MDA-MB-231 cells in a dose-dependent fashion [81]. Epigallocatechin gallate (EGCG), a key component of green tea extract, has been demonstrated to inhibit DNA methylation in vitro by binding to DNMT1, which leads to the re-expression of tumor suppressor genes [82]. Hydralazine, a weak non-nucleoside DNA methylation inhibitor [83], exerts its effects by inhibiting DNMT1 activity [84]. MG98, a 20-nucleotide antisense oligonucleotide, blocks DNMT1 translation by complementing the 3′-UTR of DNMT1 mRNA, inducing demethylation in vitro and in vivo [85]. DNA methylation is one of the most important regulatory mechanisms in epigenetics, significantly influencing gene expression and the process of alternative splicing, and thus playing a pivotal role in cancer initiation and progression. DNMTis have the potential to reverse DNA methylation abnormalities, restore the normal expression of tumor suppressor genes, and correct cancer cell dysfunctions caused by splicing dysregulation. These inhibitors not only provide new directions for cancer therapy but also offer potential therapeutic strategies for inhibiting tumor progression through epigenetic regulation of alternative splicing.

### 5.3. Bromodomain and Extra-Terminal Domain Inhibitors (BETis)

The bromodomain and extraterminal (BET) protein family, consisting of four conserved mammalian members BRD2, BRD3, BRD4, and BRDT, comprises key transcriptional regulators that form complexes with HDAC and other proteins to stimulate transcriptional activity [86]. BRD4 assembles on highly acetylated gene promoters and ‘super-enhancers’ to facilitate RNA polymerase II-mediated transcription initiation and elongation [87]. Consequently, BRD4 serves as a crucial transcriptional and epigenetic regulator, and targeting BET proteins represents an effective strategy for treating various diseases, including malignant tumors [88]. Molibresib is a BET bromodomain inhibitor that exhibits high affinity for the BD1/BD2 domains of BRD2/3/4 [87,89,90]. Following treatment with birabresib, BRD2, BRD4, and c-MYC proteins are significantly reduced, HEXIM1 protein increases, while BRD3 expression remains unchanged [91,92]. Pelabresib, a highly selective and effective BET bromodomain inhibitor, reduces the expression of BET-dependent genes in vivo and shows antitumor effects in MV-411 xenograft models; it is currently undergoing clinical trials for treating hematologic malignancies [93]. AZD5153, in clinical development, binds simultaneously to both bromodomains of BRD4 and has been confirmed to regulate MYC and HEXIM1 transcription, indicating its efficacy as an oral BET/BRD4 inhibitor for blood cancer [94]. ABBV-744, a targeted BET BD2 inhibitor, is currently undergoing Phase I clinical trials for the treatment of acute myeloid leukemia and prostate carcinoma [95]. BMS-986158 targets the acetyl-lysine binding site on the BET protein BRD, disrupting interactions with acetylated histones, leading to chromatin remodeling, thereby inhibiting tumor cell growth [96]. PLX51107 binds to the bromodomains of CBP and EP300, inhibiting CpG-induced primary chronic lymphocytic leukemia (CLL) cell proliferation [97]. BI 894999 inhibits the binding of bromodomains to acetyl-lysines on histones H3 and H4, making it valuable for research into treatments for AML and other cancers [98]. ZEN-3694, an end-bromodomain inhibitor, exhibits efficacy in models resistant to androgen signaling inhibitors (ASIs). ZEN-3694, used alongside enzalutamide (ENZ), is currently being assessed for safety and effectiveness in metastatic castration-resistant prostate carcinoma (MCRPC) through Phase Ib/IIa trials [99]. GS-5829 [100] is an orally administered small molecule BET inhibitor employed in treating solid tumors and hematologic malignancies. By interacting with the bromodomains of BET proteins, it inhibits the recruitment of positive transcription elongation factor b to AR target genes, thus suppressing their transcription [101]. FT-1101 demonstrates similar levels of inhibition of all four BET family members’ bromodomains and shows potent antiproliferative activity across multiple human leukemia, lymphoma, and multiple myeloma cell lines [102]. These agents disrupt the interaction between BET proteins and acetylated histones, thereby altering the expression of genes involved in cell cycle progression and survival. Importantly, BET proteins have also been implicated in the regulation of alternative splicing, likely through their interactions with transcriptional elongation complexes and chromatin remodelers. By interfering with this axis, BET inhibitors may restore normal splicing patterns and suppress oncogenic transcript isoforms.

The interplay between epigenetic regulation and alternative splicing presents a compelling therapeutic target space. Although several epigenetic drugs are approved or in development, their direct impact on alternative splicing is still under active investigation. Given that many splicing aberrations in cancer are mediated by epigenetic alterations—such as histone modification and DNA methylation—therapeutic interventions that modulate these marks may offer a means of restoring correct splicing decisions and reactivating silenced tumor suppressor pathways.

Future research should focus on identifying splicing events sensitive to epigenetic therapies, characterizing chromatin–splicing factor interactions, and developing combination regimens that simultaneously target chromatin and spliceosome components. These approaches hold promise not only for enhancing antitumor efficacy but also for minimizing resistance and improving clinical outcomes in patients with splicing-driven cancers.

## 6. Exploring Strategies for Targeting Alternative Splicing

Alternative splicing exhibits tissue specificity, and alterations in splicing often occur in a disease-specific manner during disease progression, indicating that modulating alternative splicing could represent a promising therapeutic strategy, particularly in cancer treatment. Currently, a variety of approaches and compounds have been developed or are under development to correct splicing defects in cancer. These strategies include spliceosome-mediated RNA trans-splicing (SMaRT), antisense oligonucleotides (ASOs), genome editing technologies such as CRISPR and TALEN, and small molecule compounds aimed at regulating alternative splicing.

### 6.1. Spliceosome-Mediated RNA Trans-Splicing

Spliceosome-mediated RNA trans-splicing (SMaRT) is an RNA-based method that enables the precise recombination of two distinct precursor mRNA molecules through exon–exon splicing [103]. This RNA trans-splicing approach requires three essential elements: the spliceosome, the target pre-mRNA transcript, and the pre-mRNA trans-splicing molecule (PTM) [104]. The PTM is exogenously provided and typically consists of: (1) a binding domain that recognizes the target intron in the endogenous pre-mRNA through base pairing, (2) an engineered intron that catalyzes the splicing reaction, and (3) a cDNA segment that encodes an alternative coding sequence. The arrangement of these components within the PTM is dictated by the position of the exon being replaced in the target mRNA, whether it is at the 5′ or 3′ end [105].

This infrequent trans-splicing process, mediated by the cellular spliceosome, was originally discovered in planarians and trypanosomes [106,107]. This discovery holds significant biological implications, as it reveals a spliceosome-mediated splicing process occurring between distinct RNA molecules within a cell. In vitro studies have shown that spliceosome-mediated RNA trans-splicing can facilitate RNA repair in primary cells and cell lines [108,109]. The initial in vivo application of the SMaRT method was conducted using a hemophilia A mouse model, where it successfully corrected genetic defects through RNA repair. This demonstrated the feasibility of using SMaRT for RNA-based therapy of genetic diseases [110]. An optimized trans-splicing system has also been reported, which involves co-expression of SMN2 trans-splicing RNA and antisense RNA that blocks downstream splicing sites of the SMN2 precursor mRNA. This system was the first to demonstrate in vivo that SMN2 trans-splicing alleviates the severity of the spinal muscular atrophy (SMA) phenotype [111,112]. Studies have also demonstrated that SMaRT can effectively reprogram Tau RNA. The cis-splicing exclusion of exon 10 can be circumvented through trans-splicing, enabling the conversion of exon 10− into exon 10+ tau RNA with an efficiency of approximately 34%. This paves the way for the novel therapeutic application of SMaRT in tauopathies and other diseases associated with abnormal alternative splicing [113]. Notably, systemic ganciclovir treatment in mice harboring cancer cells expressing the RNA trans-splicing molecule RTM44 led to a marked reduction in both tumor volume and weight, underscoring the potential applicability of RTM44-mediated cancer gene CT-SLCO1B3 across a range of malignancies [114]. Despite the unique advantages of the SMaRT technology, such as requiring only exogenous RNA, positioning it as a strong competitor to other molecular tools, trans-splicing has not yet been widely developed and still requires further exploration and a deeper understanding to enable the design of more efficient PTMs. One of the key challenges is predicting the 3D structures of both the target gene pre-mRNA and the PTM [105], as these structural features are likely to be critical determinants of trans-splicing efficiency.

### 6.2. Antisense Oligonucleotides

Antisense oligonucleotides (ASOs) are synthetic nucleic acid polymers, typically 18–30 nucleotides long, which can be single or double-stranded and have diverse chemical compositions. These molecules are employed to modulate gene expression through various mechanisms [115]. The mechanisms of action of ASOs are generally classified into two categories: one is based on RNase H1-mediated mRNA degradation, while the other involves steric hindrance induced by ASO binding, which blocks critical regions of the mRNA and affects its maturation or translation into protein [116,117]. For example, targeting miR-21 with an antisense oligonucleotide could be an effective approach to modulate miR-21 expression in colorectal cancer cells, potentially diminishing both their proliferation and migration [118]. Spinal muscular atrophy (SMA) is a neurodegenerative disorder resulting from the deletion or mutation of the survival motor neuron 1 (SMN1) gene. Studies have shown that ASOs targeting the SMN2 intronic splicing silencer can alter the amount of full-length SMN transcripts in the nervous system, leading to restoration of SMN to levels that correct SMA [119,120]. Recent studies have described a multifunctional lipofection-based approach for the systematic evaluation and screening of ASO candidates in human pancreatic cancer organoid models [121]. This diverse range of mechanisms makes antisense oligonucleotides (ASOs) an exceptionally versatile tool, enabling the selection of distinct modes of action for gene regulation based on disease type and therapeutic needs.

Currently, eight antisense drugs have been approved by regulatory agencies, most of which are focused on genetic diseases (Table 2). Nusinersin, approved in 2016, is the first drug for treating SMA [122]. Eteplirsen, developed by Sarepta Therapeutics and granted FDA approval on 19 September 2016, is utilized for the treatment of Duchenne muscular dystrophy (DMD) [123]. Inotersen, created by Ionis Pharmaceuticals and granted FDA approval on 5 October 2018, is indicated for the treatment of hereditary transthyretin-mediated amyloidosis (hATTR-PN) polyneuropathy in adults [124]. Volanesorsen, an apoCIII mRNA antisense oligonucleotide inhibitor, was created by Ionis Pharmaceuticals in collaboration with its subsidiary Akcea Therapeutics and was approved in the EU in May 2019 for the treatment of familial chylomicronemia syndrome (FCS) [125]. Golodirsen, developed by Sarepta Therapeutics and granted FDA approval on 12 December 2019, is indicated for treating Duchenne muscular dystrophy (DMD) in patients with dystrophin gene mutations by promoting the skipping of exon 53 [126]. Viltolarsen, produced by Nippon Shinyaku Co and approved by the FDA on 12 July 2020, is used to treat DMD in patients with confirmed dystrophin gene mutations by inducing exon 53 skipping [127]. Casimersen, developed by Sarepta Therapeutic Inc., received FDA approval on 25 February 2021 for the treatment of DMD in patients with confirmed mutations in dystrophin gene exon 45 [128]. On 25 April 2023, the FDA granted approval for tofersen to treat amyotrophic lateral sclerosis (ALS) associated with mutations in the superoxide dismutase 1 (SOD1) gene [129]. ASO drugs, due to their favorable pharmacokinetics allowing for dosing every few weeks, provide significant help to patients with various common and rare diseases. However, the adverse drug reactions (ADRs) and toxicity associated with ASO drugs require further attention. Currently, all approved ASOs are for rare diseases, but ASO drugs have also made some progress in treating common diseases, with some currently in long-term outcome trials [130]. Personalized assessment of patient physiological and pathological states, optimizing various dosing regimens and routes, and developing personalized treatment plans based on ASO mechanisms are hoped to gradually overcome more diseases with ASO therapies [130,131].

ASO therapeutics have made significant progress in the treatment of genetic disorders, neurodegenerative diseases, and certain rare diseases, owing to their precise gene expression modulation mechanisms. In particular, the RNase H1-mediated degradation mechanism has demonstrated clear advantages in treating certain genetic diseases. However, the design and mechanisms of ASO drugs still face challenges, particularly in accurately targeting specific mRNAs, minimizing off-target effects, and enhancing both efficacy and specificity. The innovation of ASOs lies in their ability to directly target mRNA, restoring or altering the expression of specific genes, thus offering therapeutic effects that traditional treatments cannot achieve. Nonetheless, ASOs still encounter challenges such as side effects, drug tolerance, and the need for personalized treatment strategies.

### 6.3. CRISPR–Cas9: Advancements in Genome Editing

Clustered Regularly Interspaced Short Palindromic Repeats (CRISPR) and CRISPR-associated protein 9 (Cas9) technology represents the third generation of gene editing, building on the advances of earlier methods such as Zinc Finger Nucleases (ZFNs) and Transcription Activator-Like Effector Nucleases (TALENs). These engineered nucleases create DNA double-strand breaks (DSBs) at specific genomic locations [139]. Host cells repair these breaks through non-homologous end joining (NHEJ) or homology-directed repair (HDR), which leads to insertions, deletions, and frame-shift mutations in the target DNA [140]. Due to its rapid advancement in various biological and therapeutic contexts, CRISPR–Cas9-mediated splice modulation holds immense potential for cancer treatment.

Studies have demonstrated that the CRISPR–Cas9 system can effectively modulate splicing-related defects. For instance, Foltz et al. generated induce pluripotent stem cells (iPSCs) corrected by CRISPR–Cas9 from fibroblasts of patients with retinal pigment degeneration caused by a missense mutation in the splicing factor PRPF8 [141]. CRISPR–Cas9-mediated excision of SINE-VNTR-Alu (SVA) elements rescued the transcriptional features specific to X-linked dystonia-parkinsonism and normalized TAF1 expression [142]. Removing CTG repeat expansion (CTGexp) sequences using CRISPR–Cas9 reversed abnormal cardiac splicing lesions in cardiomyocytes derived from myotonic dystrophy type 1 (DM1) patients [143]. Additionally, data from the initial Phase I clinical trial combining cancer immunotherapy with CRISPR–Cas9 indicate that engineered T cells exhibit long-term persistence, underscoring the feasibility and safety of using this technology in cancer treatment [144]. Thus, CRISPR–Cas9 proves to be an effective tool for regulating alternative splicing. However, despite its targeting specificity, off-target DNA cleavage may occur, and CRISPR–Cas9 could unintentionally produce non-selective splicing products, large genomic deletions, translocations, and inversions, which need further evaluation before clinical application [5].

The application of CRISPR–Cas9 technology in splice regulation has demonstrated unique potential, particularly in its ability to directly correct mutations that cause splicing defects at the genomic level. This provides a revolutionary approach for treating diseases associated with gene splicing defects, especially in the areas of retinal pigment degeneration, muscular atrophies, and neurodegenerative disorders. Studies using CRISPR–Cas9 to repair splicing defects and restore normal splicing patterns further highlight the technology’s promise in precision medicine, particularly in combination with cancer immunotherapy. However, its off-target effects and other potential sources of genomic instability remain significant challenges. Future research should focus on optimizing the precision and safety of this technology to lay the groundwork for its clinical application.

### 6.4. Small Molecules

Small molecules can also be employed to correct abnormal splicing and enhance disease outcomes, either by directly modulating splice factor activity or by indirectly influencing splicing mechanisms. Certain small molecules have proven effective in treating diseases like cancer. FR901464 and its methylated derivative, spliceostatin A, are potent spliceosome inhibitors [145,146] that block in vitro splicing and lead to the accumulation of pre-mRNA. They achieve this by binding to the SF3B subcomplex of the U2 small nuclear ribonucleoprotein (snRNP) within the spliceosome [147]. FR901464 triggers cell cycle arrest and degradation of genomic DNA nucleosomes in mice, demonstrating strong antitumor activity [146]. Spliceostatin A promotes apoptosis in chronic lymphocytic leukemia cells by downregulating MCl-1 [148]. Meayamycin B, an effective analog of FR901474, interferes with pre-mRNA splicing in HEK-293 cells and demonstrates strong antiproliferative activity against human lung and breast carcinoma cells [149,150]. Sudemycin E, a synthetic analog of FR901464, interacts with the SF3B1 subunit of the U2 snRNP, disrupting its role in preserving H3K36me3 modification in actively transcribed genes, leading to changes in chromatin modification and potentially contributing to cancer cell death [151]. Sudemycin D6, a stable variant of sudemycin E, exhibits strong cytotoxic effects against melanoma SK-MEL-2 cells as well as other cancer cell lines [152]. Sudemycin K, a more stable and effective derivative of sudemycin E, exhibits higher potency as an alternative splicing modulator and spliceosome assembly inhibitor in vitro [153]. Pladienolide B(PB) induces early intron retention and splicing inhibition in mRNA, leading to apoptosis in primary leukemia cells from CLL patients [154]. Herboxidiene (GEX1A), a potent polyene compound derived from the actinomycete *Streptomyces* sp. A7847 blocks pre-mRNA splicing through its interaction with SAP155, a component of the SF3b complex, and demonstrates both anti-cholesterol and antitumor properties [155,156]. The spliceosome inhibitor E7107 has shown significant inhibitory effects on xenograft models of acute myeloid leukemia from patients with spliceosome mutations [157].

Notably, focusing on SR proteins could represent the next advance in developing spliceosome inhibitors [158]. Overexpression of SRSF6 has been found to induce exclusion of exon 23 from ZO-1, contributing to its oncogenic role. The β2-adrenergic receptor agonist indacaterol, approved for treating chronic obstructive pulmonary disease, has been identified as an SRSF6 inhibitor and can suppress colorectal cancer proliferation [159]. As a CDC-like kinase (CLK) inhibitor, cirtuvivint (SM08502) blocks the phosphorylation of serine and arginine-rich splicing factors (SRSFs) and disrupts spliceosome function. In xenograft mouse models, it significantly slows the growth of gastrointestinal tumors and decreases both SRSF phosphorylation and the levels of Wnt pathway gene expression [160]. The selective protein kinase CK2 inhibitor silmitasertib (CX-4945) effectively inhibits CLKs in vitro, thereby reducing phosphorylation of SR proteins in mammalian cell lines [161]. It activates the PI3K/Akt/mTOR signaling pathway with antiproliferative effects in blood diseases [162,163]. 4bHWE affects selective splicing by decreasing phosphorylation of splicing factor SRSF1, increasing H3K36me3 levels, and altering chromatin condensation. This inhibitor exhibits antiproliferative effects on the human lung cancer cell line H1299 [164,165]. EXN407, a small molecule inhibitor targeting SRPK1, selectively inhibits VEGF isoforms involved in retinal vascular disease progression, reducing abnormal growth of leaking intraocular blood vessels [166].

In summary, small molecule inhibitors targeting spliceosome components have shown significant potential in cancer therapy. By intervening in spliceosome function and modulating the splicing process, these inhibitors affect gene expression and ultimately induce apoptosis in cancer cells. Several small molecules have already been approved and are in clinical use for the treatment of various diseases. However, challenges related to target specificity and delivery efficiency remain key obstacles to the broader clinical application of these therapeutic strategies. The growing interest in SARS-CoV-2 mRNA vaccines, due to their practicality and efficacy, has underscored the potential of mRNA cancer vaccines, which show promise for rapid, cost-effective, and scalable production, potentially advancing cancer treatment possibilities [167,168].

## 7. Concluding Remarks and Future Perspectives

The regulation of alternative splicing and epigenetics is profoundly intricate. The regulation of alternative splicing can be controlled at multiple levels: RNA, transcription, and chromatin structure and epigenetic modifications. This review initially explored the interplay among DNA methylation, histone modifications, and alternative splicing. Changes in epigenetic modifications can influence the binding affinities of related factors, subsequently affecting transcriptional elongation rates to regulate alternative splicing. Specific proteins can also interact with epigenetic modifications to recruit splicing factors and regulate splicing. Additionally, epigenetic modifications may influence splicing factors or their upstream regulatory genes, further impacting splicing outcomes. Importantly, the relationship between epigenetic modifications and alternative splicing is not unidirectional but dynamic and bidirectional. This reciprocal interaction offers a new framework for understanding how splicing dysregulation and epigenetic modifications influence each other, providing innovative insights into the intricate regulation of gene expression.

Although our understanding of the relationship between epigenetic modifications and alternative splicing remains incomplete, this field is rapidly evolving, particularly with respect to therapeutic strategies. The influence of epigenetic modifications on RNA processing has revealed novel targets for the treatment of diseases associated with aberrant alternative splicing, especially cancer. Given the widely recognized reversibility of epigenetic modifications, increasing research efforts have been directed toward the development of drugs targeting epigenetic mechanisms such as DNA methylation and histone modifications. Several small molecule inhibitors and epigenetic modulators have already been approved for clinical use or are currently undergoing clinical trials, demonstrating considerable therapeutic potential in various cancers and genetic disorders.

**Figure 5:**
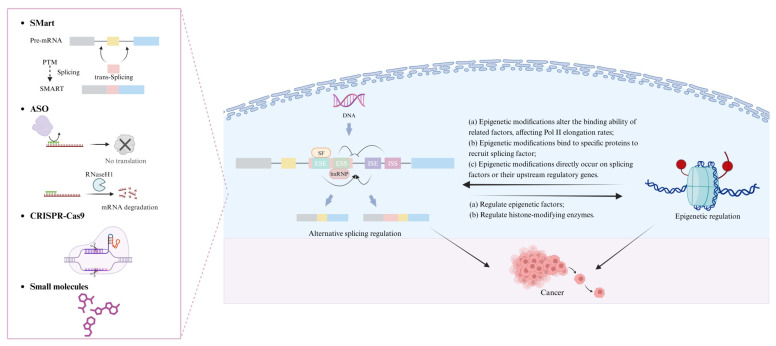
The relationship between alternative splicing, epigenetic modifications, and cancer. Epigenetic modifications and alternative splicing not only influence cancer development and progression through their respective pathways but also complexly regulate cancer biology through their interactions. Targeting alternative splicing is an emerging area in cancer research and treatment. The combined study of targeting alternative splicing and epigenetic modifications is advancing cancer therapy.

Meanwhile, therapeutic strategies targeting alternative splicing are also advancing rapidly, including spliceosome-mediated RNA trans-splicing (SMaRT), antisense oligonucleotides (ASOs), CRISPR–Cas9 gene editing technologies, and small molecules targeting splicing factors (Figure 5). These approaches not only have the potential to correct splicing abnormalities but may also act synergistically with epigenetic drugs to modulate gene expression patterns more precisely. Future research should focus on enhancing the target specificity, efficacy, and safety of these therapeutics, as challenges such as drug-related side effects and inter-individual variability remain significant concerns in clinical settings. Moreover, the development of combination therapies that integrate epigenetic drugs with splicing-modulatory agents could represent a promising direction for the treatment of various complex diseases. In summary, the development of therapeutics targeting epigenetic modifications and alternative splicing not only elucidates the multifaceted mechanisms underlying gene expression regulation but also provides a solid theoretical foundation and promising clinical prospects for the precision treatment of major diseases such as cancer.

## Figures and Tables

**Table 1:** Targeted epigenetic therapeutics.

Target	Drug	Structure	Treatment	Phase	Reference/NCT no.
HDAC	Vorinostat	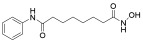	CTCL	FDA approved	[65]
	Romidepsin	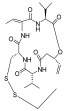	CTCL, PTCL	FDA approved	[66]
	Belinostat	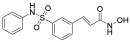	PTCL; HCC, Burkitt lymphoma, DLBCL, thymic carcinoma, MDS	FDA approved	[67]
	Panobinostat	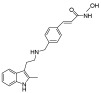	MM; thyroid carcinoma, RCC, breast cancer, AML	FDA approved	[68]
	Chidamide	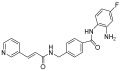	PTCL	FDA approved	[69]
	Valproic acid	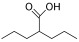	MDA, AML	II	[70]
	Abexinostat	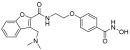	Lymphoma	I & II	[57]
	CUDC-101	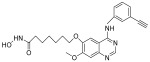	Solid tumor	I	[71]
	Pracinostat	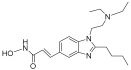	MLD	II	[59]
	Givinostat	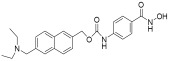	Polycythemia vera	I & II	NCT01901432
	Mocetinostat	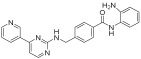	Metastatic leiomyosarcoma	II	NCT02303262
DNMT	Azacytidine	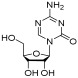	MDS, AML	FDA approved	[72]
	Decitabine	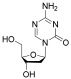	MDS, AML	FDA approved	[73]
	Guadecitabine sodium	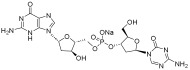	MDS, AML	II	[74]
	Aza-TdC	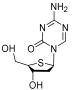	Solid tumor	I	NCT03366116
	RX-3117	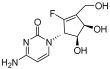	Solid tumor	I	[75]
	Epigallocatechol Gallate	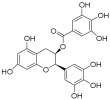	PC	II	NCT00666562
	Hydralazine	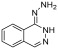	Refractory solid tumor	II	[76]
	MG98	/	Solid tumor	I	[77]
BET	Molibresib	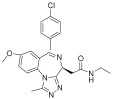	Solid tumor	I	NCT03925428
	Birabresib	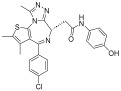	AML, DLBCL	I	NCT02698189
	Pelabresib	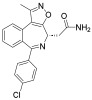	Progressive lymphoma	I	NCT01949883
	ADZ5153	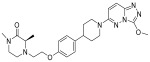	NHL, DLBCL, non-Hodgkin’s lymphoma	I	NCT03527147
	ABBV-744	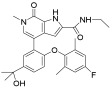	CRPC, AML	I	NCT03360006
	BMS-986158	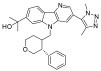	Solid tumor, lymphoma, brain tumor (pediatric)	I	NCT03936465
	PLX51107	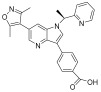	AML, myelodysplastic syndrome, MDS/MPN	I	NCT04022785
	BI 894999	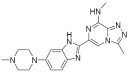	Neoplasms	I	NCT02516553
	ZEN-3694	/	MCRPC	II	NCT02705469
	GS-5829	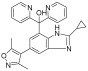	Solid tumors, lymphomas	I	NCT02392611
	FT-1101	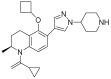	AML, MDS	I	NCT02543879

Sourced from https://clinicaltrials.gov/ (accessed on 25 December 2024).

**Table 2:** Antisense oligonucleotide drugs.

Target	Structure	ASO Drug	Dose	Treatment	Reference
Survival motor neuron-2 (SMN2) mRNA	RNA, [2′-O-(2-methoxyethyl)] (P-thio) (m5U-m5C-A-m5C-m5U-m5U-m5U-m5C-A-m5U-A-A-m5U-G-m5C-m5U-G-G)	Nusinersen	12 mg once every 4 months (IT)	Spinal muscular atrophy	[132]
Exon 51 dystrophinpre-mRNA	RNA, [P-deoxy-P-(dimethylamino)](2′,3′-dideoxy-2′,3′-imino-2′,3′-seco)(2′a ⟶ 5′)(C-m5U-C-C-A-A-C-A-m5U-C-A-A-G-G-A-A-G-A-m5U-G-G-C-A-m5U-m5U-m5U-C-m5U-A-G), 5′-[P-[4-[[2-[2-(2-hydroxyethoxy)ethoxy]ethoxy]carbonyl]-1-piperazinyl]-N,N-dimethylphosphonamidate]	Eteplirsen	30 mg/kg once weekly (IV)	Duchenne muscular dystrophy	[133]
Transthyretin (TTR) mRNA	DNA, d(P-thio)([2′-O-(2-methoxyethyl)]m5rU-[2′-O-(2-methoxyethyl)]m5rC-[2′-O-(2-methoxyethyl)]m5rU-[2′-O-(2-methoxyethyl)]m5rU-[2′-O-(2-methoxyethyl)]rG-G-T-T-A-m5C-A-T-G-A-A-[2′-O-(2-methoxyethyl)]rA-[2′-O-(2-methoxyethyl)]m5rU-[2′-O-(2-methoxyethyl)]m5rC-[2′-O-(2-methoxyethyl)]m5rC-[2′-O-(2-methoxyethyl)]m5rC) nonadeca	Inotersen	300 mg once weekly (SC)	Polyneuropathy of amyloidosis	[134]
Apolipoprotein C3	DNA, d(P-thio)([2′-O-(2-methoxyethyl)]rA-[2′-O-(2-methoxyethyl)]rG-[2′-O-(2-methoxyethyl)]m5rC-[2′-O-(2-methoxyethyl)]m5rU-[2′-O-(2-methoxyethyl)]m5rU-m5C-T-T-G-T-m5C-m5C-A-G-m5C-[2′-O-(2-methoxyethyl)]m5rU-[2′-O-(2-methoxyethyl)]m5rU-[2′-O-(2-methoxyethyl)]m5rU-[2′-O-(2-methoxyethyl)]rA-[2′-O-(2-methoxyethyl)]m5rU)	Volanesorsen	300 mg once weekly (SC)	Familial chylomicronemia syndrome	[135]
Dystrophin exon 53	RNA, [P-deoxy-P-(dimethylamino)](2′,3′-dideoxy-2′,3′-imino-2′,3′-seco)(2′a ⟶ 5′)(G-m5U-m5U-G-C-C-m5U-C-C-G-G-m5U-m5U-C-m5U-G-A-A-GG-m5U-G-m5U-m5U-C), 5′-[P-[4-[[2-[2-(2-hydroxyethoxy)ethoxy]ethoxy]carbonyl]-1-piperazinyl]-N,Ndimethylphosphonamidate]	Golodirsen	30 mg/kg once weekly (IV)	Duchenne muscular dystrophy	[136]
Dystrophin exon 53	RNA, [P-deoxy-P-(dimethylamino)](2′,3′-dideoxy-2′,3′-imino-2′,3′-seco)(2′a ⟶ 5′)(C-C-m5U-C-C-G-G-m5U-m5U-C-m5U-G-A-A-G-G-m5U-Gm5U-m5U-C)	Viltolarsen	80 mg/kg once weekly (IV)	Duchenne muscular dystrophy	[137]
Dystrophin exon 45	RNA, [P-deoxy-P-(dimethylamino)](2′,3′-dideoxy-2′,3′-imino-2′,3′-seco)(2′a ⟶ 5′)(C-A-A-m5U-G-C-C-A-m5U-C-C-m5U-G-G-A-G-m5U-m5U-C-Cm5U-G), 5′-[P-[4-[[2-[2-(2-hydroxyethoxy)ethoxy]ethoxy]carbonyl]-1-piperazinyl]-N,N-dimethylphosphonamidate]	Casimersen	30 mg/kg once weekly (IV)	Duchenne muscular dystrophy	[128]
Superoxide dismutase type 1	DNA, d([2′-O-(2-methoxyethyl)]m5rC-sp-[2′-O-(2-methoxyethyl)]rA-[2′-O-(2-methoxyethyl)]rG-sp-[2′-O-(2-methoxyethyl)]rG-[2′-O-(2-methoxyethyl)]rA-sp-Tsp-A-sp-m5C-sp-A-sp-T-sp-T-sp-T-sp-m5C-sp-T-sp-A-sp-[2′-O-(2-methoxyethyl)]m5rC-[2′-O-(2-methoxyethyl)]rA-sp-[2′-O-(2-methoxyethyl)]rG-[2′-O-(2-methoxyethyl)]m5rC-sp-[2′-O-(2-methoxyethyl)]m5rU)	Tofersen	100 mg/kg once every 3 week3(IT)	Amyotrophic lateral sclerosis	[138]

IT, intrathecal; SC, subcutaneous; IV, intravenous.

## Data Availability

Not Applicable.

## Abbreviations

The following abbreviations are used in this manuscript:

SMaRT	spliceosome-mediated mRNA trans-splicing
ASO	antisense oligonucleotides
ncRNAs	non-coding RNAs
CTCF	CCCTC-binding factor
MeCP2	methyl-CpG-binding protein 2
HP1	heterochromatin protein 1
MRG15	MORF-related gene 15
PTB	polypyrimidine tract-binding
TSA	trichostatin A
HDAC	histone deacetylase
VPA	valproic acid
AML	acute myeloid leukemia
DNMTs	DNA methyltransferases
DNMTis	DNMT inhibitors
EGCG	epigallocatechin gallate
BET	bromodomain and extraterminal
CLL	chronic lymphocytic leukemia
ASIs	androgen signaling inhibitors
ENZ	enzalutamide
MCRPC	metastatic castration-resistant prostate carcinoma
PTM	pre-mRNA trans-splicing molecule
SMN1	survival motor neuron 1
hATTR-PN	hereditary transthyretin-mediated amyloidosis
ALS	amyotrophic lateral sclerosis
SOD1	superoxide dismutase 1
ADRs	adverse drug reactions
ZFNs	zinc finger nucleases
TALENs	transcription activator-like effector nucleases
DSBs	DNA double-strand breaks
NHEJ	non-homologous end joining
HDR	homology-directed repair
iPSCs	induced pluripotent stem cells
SVA	SINE-VNTR-Alu
CTGexp	CTG repeat expansion
DM1	myotonic dystrophy type 1
CLK	CDC-like kinase
SRSFs	serine and arginine-rich splicing factors
